# A comparative analysis of lipoprotein transport proteins: LolA and LolB from * Vibrio cholerae* and LolA from *Porphyromonas gingivalis*

**DOI:** 10.1038/s41598-023-33705-y

**Published:** 2023-04-24

**Authors:** Deepika Jaiman, Raghavendra Nagampalli, Karina Persson

**Affiliations:** 1 Umeå Centre for Microbial Research (UCMR), Umeå, Sweden; 2 Department of Chemistry, Umeå University, 90187 Umeå, Sweden

**Keywords:** Microbiology, Structural biology

## Abstract

In Gram-negative bacteria, N-terminal lipidation is a signal for protein trafficking from the inner membrane (IM) to the outer membrane (OM). The IM complex LolCDE extracts lipoproteins from the membrane and moves them to the chaperone LolA. The LolA-lipoprotein complex crosses the periplasm after which the lipoprotein is anchored to the OM. In γ-proteobacteria anchoring is assisted by the receptor LolB, while a corresponding protein has not been identified in other phyla. In light of the low sequence similarity between Lol-systems from different phyla and that they may use different Lol components, it is crucial to compare representative proteins from several species. Here we present a structure–function study of LolA and LolB from two phyla: LolA from *Porphyromonas gingivalis* (phylum bacteroidota), and LolA and LolB from *Vibrio cholerae* (phylum proteobacteria). Despite large sequence differences, the LolA structures are very similar, hence structure and function have been conserved throughout evolution. However, an Arg-Pro motif crucial for function in γ-proteobacteria has no counterpart in bacteroidota. We also show that LolA from both phyla bind the antibiotic polymyxin B whereas LolB does not. Collectively, these studies will facilitate the development of antibiotics as they provide awareness of both differences and similarities across phyla.

## Introduction

**Figure 1:**
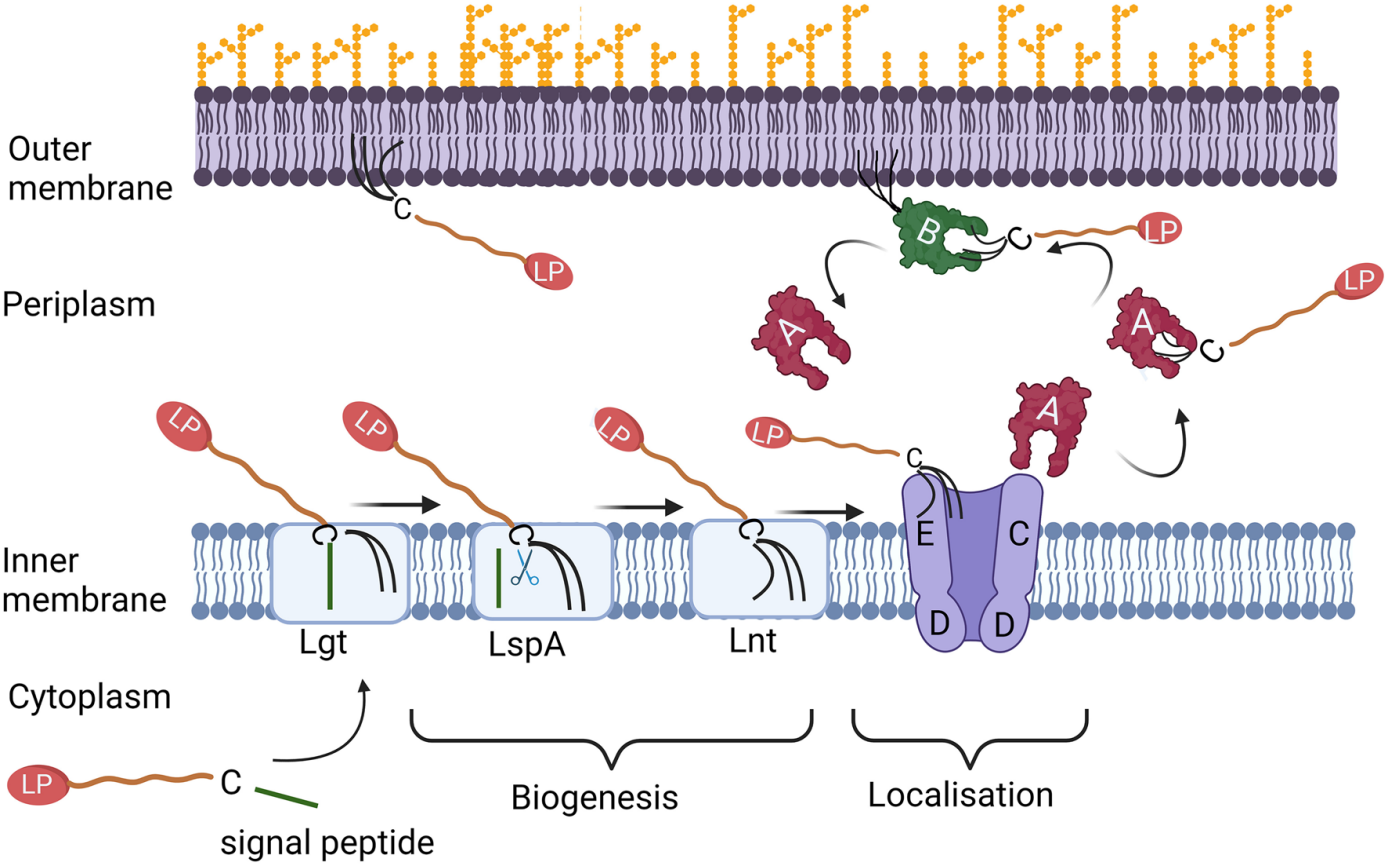
Lipidation and transport of lipoproteins. The prelipoprotein (LP) is transported over the IM where it is diacylated by the enzyme Lgt. Next the signal peptide is removed by the signal peptidase LspA. A third acyl chain is added to the N-terminal cysteine (in γ-proteobacteria) by the enzyme Lnt. The lipoprotein recognized by the LolCDE complex, is transported to the chaperone protein LolA, which in turn will deliver it to LolB at the OM (in γ-proteobacteria). The figure was created with BioRender.com.

Gram negative bacteria are surrounded by both an inner membrane (IM) and an outer membrane (OM) that are separated by the peptidoglycan-containing periplasm. Both membranes consist of double layers of phospholipids and also contain a large number of associated and integral proteins. The proteins that span the OM are generally porins, composed of β-barrels, through which a large number of molecules can be passively transported^1^. Furthermore, the outer leaflet of the OM contains glycolipids and lipopolysaccharides, which make up a large proportion of the molecules the bacterium presents to the outside and also act as a protective barrier. Proteins that span the IM mainly consist of transmembrane helices and can have various functions, such as the transport of unfolded proteins from the cytoplasm^2,3^, selective exchange of metabolites^4^ or energy generation^5^. Both membranes additionally contain lipoproteins that are soluble proteins attached to the lipid bilayer through acyl chains that are covalently linked to their N-termini. Initially they are synthesized as prelipoproteins in the cytoplasm, and via their signal peptide directed over the IM through the Sec- or Tat translocon^3^. While attached to the IM with the signal peptide, two acyl chains are transferred to the sulfhydryl of a conserved cysteine. This reaction is catalysed by the enzyme diacylglyceryl transferase (Lgt)^6^. Next, the signal peptide is cleaved off by signal peptidase II (LspA) leaving the acylated cysteine as the first residue—the two acyl chains now function as the membrane anchor^7^. In γ-proteobacteria such as *Vibrio cholerae* or *Escherichia coli*, also the free amino group of the cysteine will be acylated, a reaction that is catalysed by the enzyme apolipoprotein *N*-acyltransferase (Lnt)^8^, resulting in the mature lipoprotein with three acyl chains. Lnt has not been identified in all bacteria, for instance in bacteroidota, which suggests that their lipoproteins may be predominantly diacylated (Fig. 1).

Whether the lipoprotein is to remain anchored to the IM or transported to the OM is determined by the motif directly downstream of the acylated cysteine. For proteins that are destined to the OM a system called localization of lipoproteins (Lol) is required^9,10^. When the lipoprotein has the appropriate motif, it will be recognized by the ABC transporter complex, LolCDE, that spans the IM. A soluble protein with chaperone function, LolA, binds to a periplasmic part of LolC while waiting for the LolCDE complex to extract a lipoprotein from the membrane. Next LolA changes its binding partner to the acyl part of the lipoprotein and transports it over the periplasm as a soluble chaperone-cargo complex. In β- and γ-proteobacteria, there is a recipient protein, LolB, which itself is acylated and anchored to the OM. LolB receives the lipoprotein from LolA and transfers the lipid moity to the inner leaflet of the OM^9,10^ where most of the lipoproteins in proteobacteria end up. On the contrary, all other bacteria including those of the bacteroidota phylum, such as *Porphyromonas gingivalis*, do not have the recipient protein LolB and how the lipoproteins are introduced to the outer membrane is not known. Another intriguing feature in bacteroidota is that they have a higher proportion of lipoproteins linked to the outer leaflet, facing the extracellular milieu^11^, indicating another unknown mechanism, which in this case moves lipoproteins across the outer membrane.

Lipidation and transport of lipoproteins are critical steps to maintain the complexity and function of the bacterial membranes and is crucial for the survival of the bacteria. Therefore, all these associated steps can serve as potential targets for the development of novel antibacterial substances. For instance, earlier studies demonstrating the inhibitory effect on *E. coli* LolCDE^12^ have shown significant promise in developing new anti-microbials^13^. Furthermore, LolA (of *E. coli*) has been proposed to function as a transporter of the antibiotic polymyxin^14^ and it has also been debated whether or not LolA binds the inhibitory compound A22^15^. Crystal structures of LolA have previously been solved from mainly γ-proteobacteria such as *Pseudomonas aeruginosa*, *E. coli* and *Yersinia pestis*^16,17^. In addition to this, LolA from *Bacteroides uniformis* (phylum bacteroidota) and *Thermus thermophilus* (phylum deinococcota) are also present in the protein data bank but have not yet been described in any publication. Additionally, LolA from *E. coli* (LolA-EC) has also been studied in complex with LolC both using crystallography and cryo-electron microscopy^18,19^. These studies were recently supplemented with the X-ray structure of *E. coli* LolA in complex with a triacylated peptide^20^. Comparatively, LolB, which serves as an attractive drug target against *Vibrio parahaemolyticus*^21^, is less studied, with only the *E. coli* crystal structure being published^17^.

In this report we describe the crystal structures of Lol proteins representing bacteria of two different phyla: LolA and LolB from *V. cholerae* (proteobacteria) and LolA from *P. gingivalis* (bacteroidota). We furthermore analyse the interaction between these Lol proteins and with polymyxin B and A22. By the contribution of this study and, in combination with sequence analysis of several other species of bacteria, we can begin to understand the differences and similarities between proteins that are expressed by bacteria from different phyla. Collectively the results discussed in this study pave the way for future drug discovery projects.

## Results and discussion

### Overall architecture of LolA-PG and LolA-VC

Full length LolA from *P. gingivalis* (LolA-PG) and *V. cholerae* (LolA-VC), corresponding to the mature forms of the proteins, were expressed in *E. coli*. After protein purification they were crystallized, and data were collected. The structure of LolA-PG was solved using single anomalous dispersion (SAD) on selenomethionine (SeMet) labelled protein whereas the structure of LolA-VC was determined with molecular replacement using a model obtained from Alphafold^22^. The models were refined to 1.7 and 1.8 Å respectively. Data processing and refinement statistics are presented in Supplementary Table S1.

**Figure 2:**
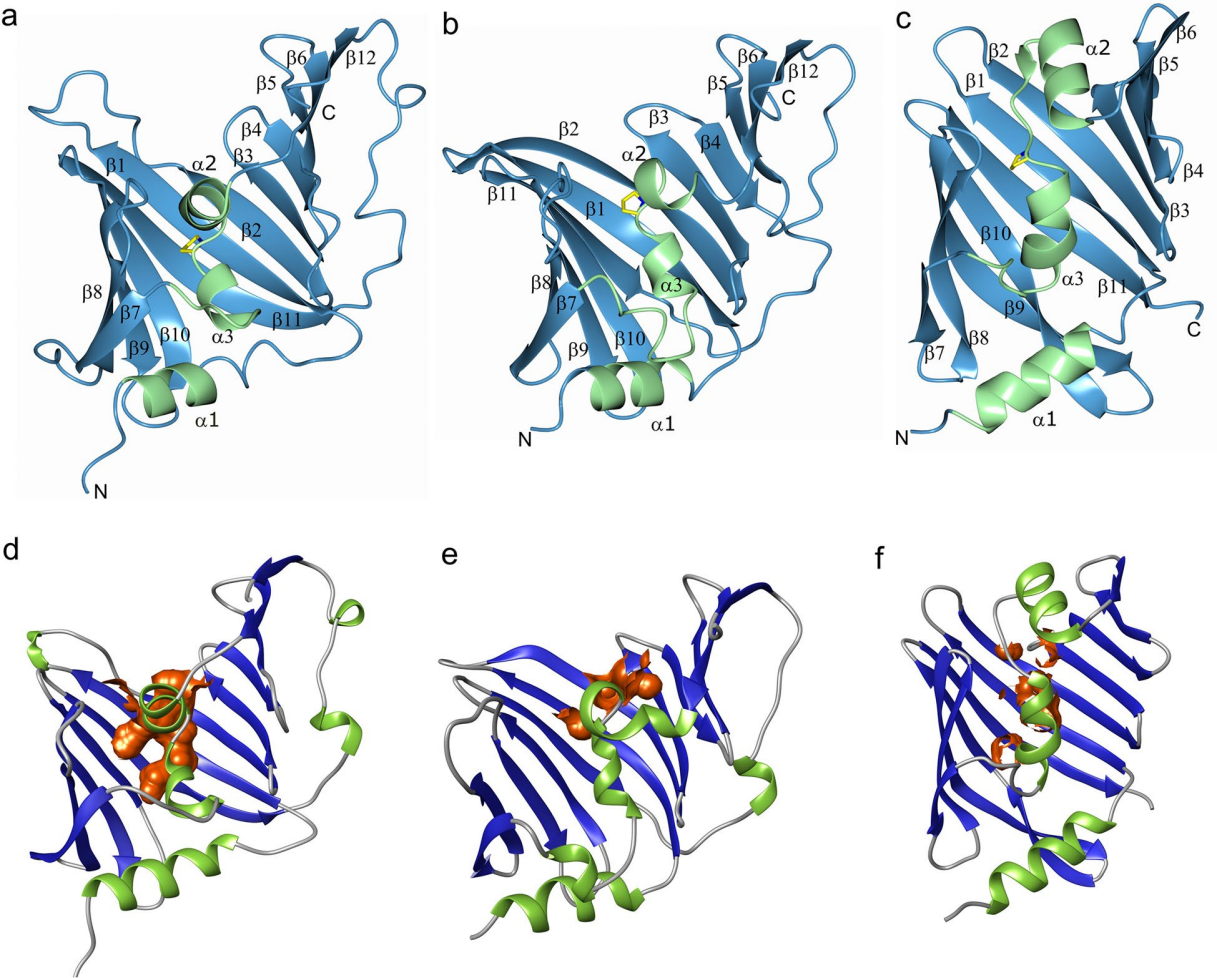
The overall structures of LolA and LolB. (**a**) LolA from *P. gingivalis* (LolA-PG), (**b**) LolA (LolA-VC) and (**c**) LolB from *V. cholerae* (LolB-VC). The LolA-PG and LolA-VC structures (ribbon drawings) fold as a 12 β-strand open barrel with three α-helices. LolB-VC comprises an 11 stranded open β-barrel with three helices. A conserved proline located between helix α2 and α3 is shown as a stick model in all three proteins. The β-strands and loops are depicted in blue and the helices in green. (**d–f**) The solvent accessible part of the binding clefts were calculated with CASTp^48^ and are illustrated in orange. The cleft in LolA-PG is the most accessible with a volume of 36 Å^3^. The clefts of LolA-VC and LolB-VC are more scattered and comprise 0.15 and 11 Å^3^ respectively.

LolA from both *P. gingivalis* and *V. cholerae* consist of a large curved β-sheet comprising twelve β-strands (Fig. 2a,b). Eleven of the strands are antiparallel whereas the last strand, β12, runs parallel to β6 on the flank of the β-sheet. β12 is connected to β11, in the center of the sheet, via a 20 amino acid (aa) extended segment that runs on the convex side of the β-sheet.

The electron density for both LolA structures is generally good, but weaker in some loops, especially between strands β8 and β9 of LolA-VC which constitutes part of the opening of the binding cleft. The equivalent loop in LolA-PG is well ordered, probably due to stabilizing crystal contacts. Additional small differences are found in other loops or turns that connect the β-strands. The concave surface forms a mainly non-polar cleft which is shielded from solvent by two helices, α2 and α3 (a 3_10_ helix). The N-terminal helix, α1, forms the floor of the cleft. The non-polar cleft can interact either with the periplasmic loop of LolC, when uncharged, or the acyl chains of a lipoprotein during protein transportation^18,20^. The helical segment that fills the hydrophobic cavity is comprised of 22 aa in both LolA-PG and LolA-VC but the different position of the helices results in different accessibility to the clefts. In LolA-PG the cleft is continuous and relatively open (36 Å^3^) as compared to the scattered cleft in Lol-VC which is occluded and only expose 0.15 Å^3^ (Fig. 2d,e).

### Overall architecture of LolB-VC

The mature but non-acylated form of LolB from *V. cholerae* (LolB-VC), was overexpressed in *E. coli*, purified and crystallized. Diffraction data were collected, and the LolB-VC structure was determined using molecular replacement. The model was refined to 1.46 Å resolution (Supplementary Table S1). The fold of LolB-VC is similar to LolA, with a large curved β-sheet consisting of eleven β-strands that folds into a hydrophobic cavity filled with two short helices. In contrast to LolA, LolB-VC does not have the long-extended segment running on the convex side of the sheet nor a 12th β-strand (Fig. 2c). Furthermore, the helical segment that fills the hydrophobic cavity of LolB-VC is slightly longer, approximatively 30 aa, leaving 11 Å^3^ of the cleft accessible in this crystal structure (Fig. 2f).

### Comparative structural analyses

LolA-VC is structurally very similar to other known LolA proteins, especially those from γ-proteobacteria. A DALI search^23^ using LolA-VC identified LolA from *E. coli*, *Y. pestis* and *P. aeurginosa* (PDB codes 1ua8, 4ki3 and 2w7q)^16,17^ as the closest structural relatives (Z-scores 25–22). When using LolA-PG as the search model, LolA from *P. aeurginosa* (PDB code 2w7q)^16^ was recognized as the structurally most similar (Z-score 19, sequence identity 13%). LolA from *B. uniformis* (LolA-BU) (PDB code 4mxt), which also originates from the bacteroidota phylum resulted in a Z-score of 18, albeit the sequence identity is higher, 24%. A pairwise structural alignment to compare LolA-VC and LolA-PG, using the structural alignment tool in the protein data bank (rcsb.org), resulted in an r.m.s.d. of 2.8 Å calculated on 176 aa and a sequence identity of 18%.

A sequence analysis was performed on the proteins described (Supplementary Fig. S1a). Within each phylum, proteobacteria (class γ-proteobacteria) and bacteroidota, the sequence similarity is high. When proteins from both phyla were analyzed together, only three identical aa are found: two phenylalanines (on β1 and β3 respectively) contributing to the hydrophobic environment in the binding cleft. The third conserved residue is a proline (Pro120 in LolA-PG and Pro105 in LolA-VC) that creates the bend between the α2 and α3 helices, that fill the hydrophobic cavity (Fig. 2a,b).

**Figure 3:**
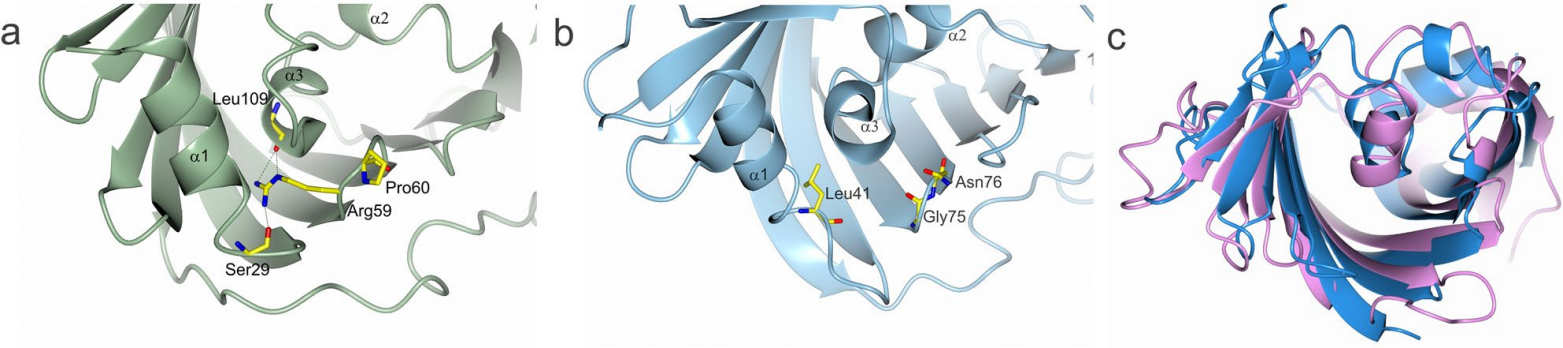
A comparison of the binding clefts in LolA. (**a**) In γ-proteobacteria, such as *V. cholerae*, an Arg-Pro motif (Arg59 and Pro60 in LolA-VC) is conserved. In *E. coli* the motif has been shown to be important for the interaction with LolB and the transfer of lipoproteins. With no binding partner bound in the cleft of LolA-VC Arg59 hydrogen bonds to main chain atoms at the end of α1 and of α3. (**b**) In LolA-PG the position equivalent to Arg59 is occupied by Gly75. The space that is filled by the Arg59 side chain in LolA-VC is filled with Leu41 in LolA-PG. (**c**) Overlay of LolA-EC in blue and LolA-PG in pink showing the similar size and shape of their cavities.

An Arg-Pro motif located on the turn between β2 and β3 is conserved in γ-proteobacterial LolA (Arg59, Pro60 in LolA-VC). There, the arginine side chain is facing the interior of the protein where it interacts with main chain carbonyls of α2 (Fig. 3a). Mutational studies in *E. coli* have shown that the arginine is important for lipoprotein transfer to LolB^24^. When the arginine was mutated to leucine the delivery of lipoprotein to LolB was negatively affected however the interaction between LolA (without bound lipoprotein) and LolB became stronger^20^. The recent structure of LolA-EC in complex with a triacylated peptide indeed conferred that the bound acyl chains and protein obtain different conformations when the arginine is mutated to leucine, and as a consequence the delivery of lipoprotein to LolB is altered^20^. The Arg-Pro motif is not present in LolA-PG or in LolA-BU. Instead, they both have a glycine located at the corresponding position (Gly75 in LolA-PG). The equivalent space of the arginine side chain is instead filled with the hydrophobic Leu41 in LolA-PG (Phe43 in LolA-BU). This is an interesting difference considering that a recipient LolB protein has not been identified in bacteroidota, hence the function of these LolA proteins must rely on other patterns of interaction (Fig. 3a,b). It should be added that when the sequence analysis was extended to include LolA also from other classes of proteobacteria it is established that the Arg-Pro motif is also found in β-proteobacteria (*Neisseria meningitidis*) but not ε-proteobacteria (*Helicobacter pylori*), hence the motif is not universal even among proteobacteria. Furthermore, there is a difference in the theoretical isoelectric point for the two proteins in this study, 4.8 for LolA-VC and 9.3 for LolA-PG calculated on the mature proteins. This may reflect differences in milieu and interaction partners. It is also interesting to speculate if LolA-PG and other bacteroidota LolA proteins really have evolved to bind diacylated substrates, as has been assumed since an *lnt* gene has not been identified. By comparing the Lol-PG binding cleft to LolA-EC (Fig. 3c) there are no obvious differences indicating that only two acyl chains should be accommodated by the bacteroidota protein. Indeed, it has been experimentally shown that the *P. gingivalis* protein PG1828 and the *Bacteroides fragilis* protein BF1333 are triacylated^25,26^, but there is to our knowledge no studies that describe if this is the most common lipidation in bacteroidota or not. As the sequence homology between the different phyla is very low it is possible that an enzyme that adds a third acyl chain, just like Lnt in γ-proteobacteria, does exist—but has not yet been identified. Hence, we hypothesize that some bacteroidota's lipoproteins may be triacylated, just like in γ-proteobacteria.

LolB, is unlike LolA, itself a lipoprotein and is acylated at residue Cys27 and bound to the inner leaflet of the OM. There it receives lipoproteins from LolA and anchors them to the membrane. A Dali search using the LolB-VC structure unsurprisingly identified LolB from *E. coli* (LolB-EC) as the closest structural relative with a Z-score of 26 and an r.m.s.d of 1.4 Å with 31% sequence identity of 172 aligned residues^17^. Further down the list of other putative lipoprotein sorting proteins, most likely LolA proteins, are found, with Z-scores 12–13. For LolB-EC it has been shown that a leucine (Leu68) is important for its function to accept lipoprotein from LolA and target them to the membrane^27^, Indeed, LolB-VC exhibits a leucine (Leu63) in the same position, an indication of a conserved function. Interestingly, both LolB proteins have a proline positioned between helix α2 and α3 as previously described for LolA (Fig. 2c). An interesting difference between LolB-EC and LolB-VC is first of all the difference in theoretical pI, 8.7 for LolB-EC and 5.1 for LolB-VC. This may also be reflected by the more negative surface potential of the helices that fill the binding cavity of LolA-VC. Further, the main structural difference is that strands β9 and β10 each are two residues longer in LolB-VC (Supplementary Fig. S2).

### The interaction between LolA and LolB

**Figure 4:**
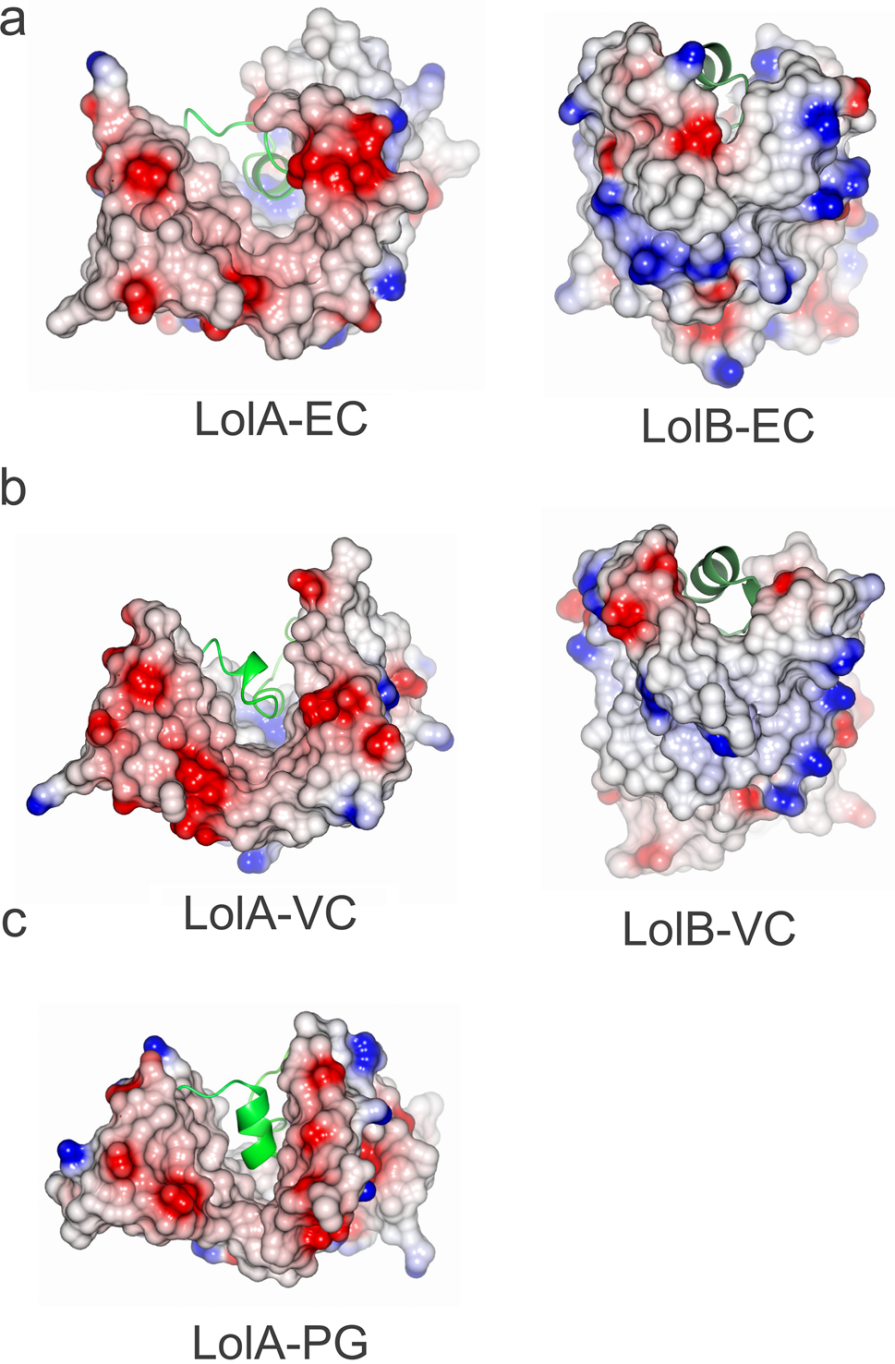
Electrostatic representation of the opening of the hydrophobic cavity of LolA and LolB from different bacteria. (**a**) The hydrophobic cavity of LolA-EC is lined with negatively charged residues. The cavity of LolB-EC is on the other hand predominantly lined with positively charged residues. (**b**) LolA-VC and LolB-VC have similar surface charge distribution. (**c**) The hydrophobic cavity of LolA-PG is similarly lined with negatively charged residues. For clarity the helices filling the cavities are depicted as ribbons in green.

The transfer of acylated proteins between LolA and LolB is proposed to occur through a mouth-to-mouth interaction^28^ where the opening of their respective clefts dock and allow the acyl chains of the transported lipoprotein to slide from LolA to LolB (Fig. 1). It was originally described for the *E. coli* system that negatively charged residues predominantly reside on the LolA side, matched by positively charged residues on LolB^29^. An electrostatic surface representation of LolA-VC and LolB-VC indeed show that the residues lining the LolA-VC cleft is mainly negatively charged whereas LolB-VC has a ring of positively charged residues around the opening (Fig. 4a,b). We used isothermal titration calorimetry (ITC) to investigate if the recombinant proteins LolA-VC and LolB-VC can interact without the presence of lipoprotein as has been reported for *E. coli*. The measurements showed that the two proteins indeed interact, with a dissociation constant (Kd) measured to 30.2 µM (Table 1, Supplementary Fig. S3, Supplementary Table S2). This is similar to the Kd of 30.6 µM that previously was reported for the interaction between LolA and LolB of *E. coli*^29^. LolA-PG, for which a LolB partner has not been identified, showed no significant interaction to LolB-VC despite its predominantly negatively charged surface at the cleft opening (Fig. 4c, Supplementary Fig. S3, Table 1, Supplementary Table S2).

**Table 1:** ITC data for combinations of LolA, LolB, polymyxin and A22.

	Kd in µM
LolA-VC + LolB-VC	30.2 ± 3.8
LolA-PG + LolB-VC	No binding
LolA-PG + polymyxin B	13.8 ± 0.7
LolA-VC + polymyxin B	56 ± 6.5
LolA-PG + A22	681 ± 44.1

### Interactions between LolA, LolB and polymyxin B

**Figure 5:**
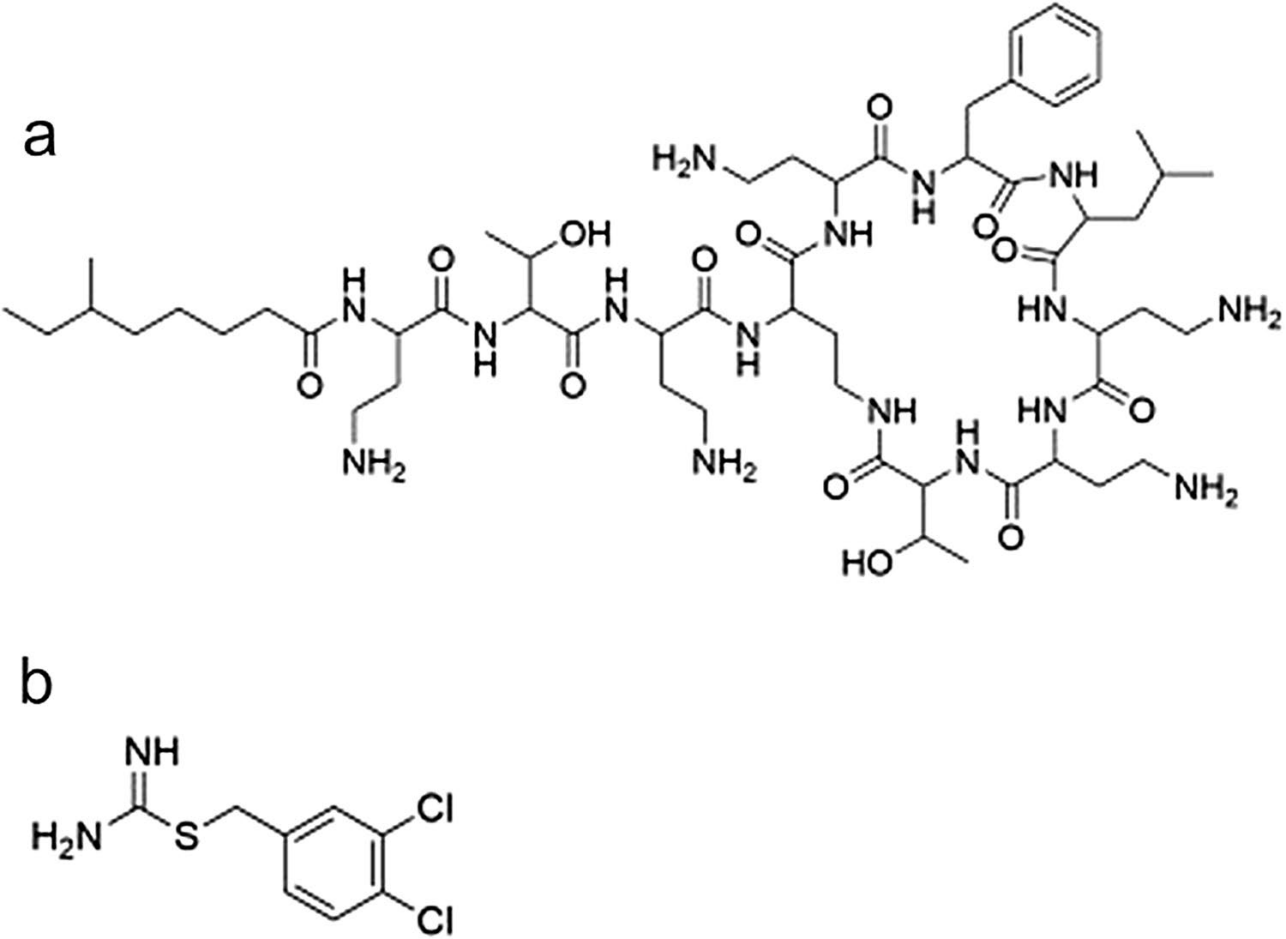
Chemical structures of the antibacterial molecules polymyxin B and A22. (**a**) The antibiotic polymyxin B and (**b**) the inhibitor A22.

Polymyxins are cyclic cationic lipopeptide antibiotics (Fig. 5a) that through electrostatic attraction can interact with lipid A of the lipopolysaccharides. As a result, the OM is disrupted, and after passing through the periplasm, the IM can also be damaged^30^. In order to uncover the mechanism of polymyxin B transport from the OM to the IM, a molecular dynamics simulations study suggested that LolA and LolB are responsible for binding and transporting the molecule^14^. The idea was derived from the observation that polymyxin has a lipid tail that could theoretically bind LolA and LolB like the acyl chains of lipoproteins.

We investigated this hypothesis experimentally by ITC and could conclude that LolA from both *V. cholerae* and *P. gingivalis* bind polymyxin B. LolA-PG shows higher affinity for polymyxin with a Kd of 14 µM compared with 56 µM for LolA-VC. When such experiment was performed on LolB-VC, the results were not conclusive. For LolB the high Kd in millimolar range, in addition to solubility issues of polymyxin B, affected the reproducibility of the experiments and therefore the analysis (Table 1, Supplementary Table S2, Supplementary Fig. S4). Despite the relatively high affinity of LolA to polymyxin B, previous studies have shown that *P. gingivalis* is resistant to polymyxin B concentrations up to 200 µg/mL. In this regard a lipid A 4ʹ phosphatase (PGN_0524) plays a crucial role since it removes a phosphate group from lipid A and makes the surface less negatively charged and hence less prone to attract the cationic polymyxin^31^. In strains carrying this enzyme it is likely that very little polymyxin enters the periplasm where LolA is active. However, this opens the prospects for development of combinations of strong physical OM disruptors of *P. gingivalis* together with novel polymyxin-like compounds that can be used synergistically against *P. gingivalis* infections. This is of high importance since *P. gingivalis* also has increased minimum inhibitory concentration (MIC) values for antibiotics such as tetracyclines, macrolides, lincosamide and fluoroquinolones and there is a fear that resistance can be transferred from other bacteroidota and prevotella species^32^. Also *V. cholerae* exhibits different sensitivity to polymyxin depending on strain. Whereas some classical strains are very sensitive to the antibiotic (MIC 0.15 μg/mL), El Tor strains express three enzymes that add a glycine to Lipid A resulting in a bacterial surface less prone to bind polymyxin B and yielding a MIC of 100 μg/mL^33^.

### Interactions between LolA, LolB and A22

It was discovered that lipoprotein trafficking in *E. coli* is disrupted by the small antibacterial compound MAC13243, or its hydrolysis product A22^34^ (Fig. 5b). In in vitro assays it was shown that these compounds induce release of lipoproteins from LolA^20^ whereas it is claimed that in vivo targets of these compounds are not LolA or LolB but instead the Mre system^35^. In this study we used ITC to clarify if A22 interacts with recombinant LolA or LolB, when they are not charged with lipoproteins. We found that LolA and LolB from *V. cholerae* have Kds in the millimolar range, that could not be measured with high confidence due to the low solubility of A22 (Supplementary Table S3, Fig. S5). On the contrary, LolA-PG has higher and more reproducible affinity for A22 and the Kd was measured to 680 µM. Hence, LolA and LolB from both γ-proteobacteria and LolA from bacteroidota bind A22 albeit with very low affinity, which is in line with previous studies.

## Conclusions

The outer membrane of Gram-negative bacteria is formed and maintained by lipoproteins, which also provide a barrier against antibiotics. The Lol system is crucial for properly delivering these lipoproteins over the periplasm to the OM and is essential for bacterial survival. The proteins that are part of the Lol system therefore are promising targets for future antibacterial substances. Since there are many different bacteria included in the term “Gram-negative”, it is important to study proteins that perform the same task but originate from different phyla. Here we have solved the crystal structures of LolA and LolB from *V. cholerae*, belonging to the phylum proteobacteria (class γ-proteobacteria), and LolA from the phylum bacteroidota. Although LolA from the two bacteria have very low sequence identity, the structures are still very similar. Nevertheless, there are important differences that one must be aware of if LolA is to be used as a drug target. LolA from *P. gingivalis* exhibits, for example, a binding pocket that is more accessible to the exterior, whereas LolA from *V. cholerae* and other γ-proteobacteria have an Arg-Pro motif that is important for transferring the lipoprotein cargo to LolB. LolA from bacteroidota does not have this motif, nor does it have a known LolB partner. Our measurements very well show that there is no cross-reaction between LolA from *P. gingivalis* and LolB from *V. cholerae*. Further, by comparing the binding clefts of LolA-EC and LolA-PG we cannot see any structural evidence that LolA from bacteroidota only should accommodate two acyl chains whereas γ-proteobacteria binds three. It is important not to rule out that bacteroidota may have the missing components of the lipidation and lipoprotein transport system (Lnt and LolB). A thorough analysis of the *P. gingivalis* genome using bioinformatics and analyzing alphafold models can perhaps find both an apolipoprotein *N*-acyltransferase and a protein with LolB function. Intriguingly, a recent publication revealed that LolA from *Caulobacter vibrioides* (α-proteobacteria), shares structural characteristics with LolB, implying that this protein may be responsible for both transport and the insertion of lipoproteins^36^. Hence, it is possible that lipoprotein transport is more diverse than it was previously believed. Finally, we have also shown that LolA from both *P. gingivalis* and *V. cholerae* bind the antibiotic polymyxin B, just as had been suggested by molecular modelling. Finally, we have shown that LolA and LolB bind to the inhibitor A22, but with such low affinity that the molecule does not constitute a promising starting structure to develop further.

## Methods

### Cloning, overexpression and purification of LolA

The *lolA* gene from *Porphyromonas gingivalis* (GenBank CP025930, *pgn_0486*) was PCR amplified from genomic DNA of strain ATCC 33277. Primers were designed not to include the signal peptide residues 1–26. The *lolA* PCR product was digested with *NcoI* /*XhoI*, and ligated into equivalent sites of pET-His1a, in-frame with a tag with sequence MKHHHHHHPMSDYDIPTTENLYFQGAM followed by LolA residues 27–215. Selenomethionine (SeMet)-labelled LolA-PG was expressed in *E. coli* BL21(DE3) by growing the cultures in minimal media supplemented with 0.4% glucose and 50 μg/mL kanamycin at 37 °C. At an OD600 ~ 0.4, 100 mg/L each of lysine, threonine, phenylalanine and 50 mg/L each of leucine, isoleucine, valine, proline and SeMet were added^37^. After 15 min, protein expression was induced with 0.5 mM IPTG. The cultures were grown for 16 h at 24 °C. Cells were harvested by centrifugation and the pellets stored at − 80 °C until further use. The cell pellets were resuspended lysis buffer (50 mM sodium phosphate buffer pH 7.6, 0.3 M NaCl and 10 mM imidazole supplemented with 2 mM palmitic acid, 1% triton-X100) and sonicated on ice. The lysate was centrifuged (63,000×*g* for 20 min). The resulting supernatant was passed over a column packed with His60 Ni-resin (Takara). The column was washed with lysis buffer containing 30 mM imidazole after which the protein was eluted with the same buffer containing 0.3 M imidazole. Next, the histidine tag was removed by incubation with ~ 1% (w/w) TEV protease over night at + 4 °C. After buffer exchange (50 mM sodium phosphate pH 7.6, 0.2 M NaCl) the protein solution was passed over the affinity column again. The flow through and wash fractions were collected and concentrated. The cleaved protein was further purified on by gel filtration (HiLoadTM 16/60 Superdex™200 prep-grade column (GE Healthcare)) equilibrated with the same buffer. Fractions containing the peak of interest were concentrated (20 mM HEPES pH 7.5).

The *lolA* gene from *V. cholerae* (WP_001045828.1) was PCR amplified from genomic DNA of strain A1552 and cloned into pET-His1a using restriction sites *NcoI* and *EcoRI.* The signal peptide (residue 1–16) was not included in the construct. Hence the final construct consisted of the tag described above and LolA residues 17–198. His-tagged LolA-VC was expressed in *E. coli* BL21 (DE3) by growing the cultures in LB media supplemented 50 μg/mL kanamycin at 37 °C. At an OD600 ~ 0.6, protein expression was induced with 0.5 mM IPTG. The cultures were grown for 16 h at 18 °C. Cells were harvested by centrifugation and the pellets stored at − 80 °C until further use. LolA-VC was purified as described as above and stored (20 mM HEPES pH 7.6, 0.2 M NaCl).

The *V. cholerae* A1552 *lolB* gene (WP_001961643.1) was similarly amplified and cloned into pET-His1a using restriction sites *NcoI* and *EcoRI*. The signal peptide (residues 1–26) was not included in the construct. The construct is comprised of the same tag as described before followed by LolB residues 27–211. His-tagged LolB-VC was expressed in *E. coli* BL21(DE3) by growing the cultures in LB media supplemented 50 μg/mL kanamycin at 37 °C. At an OD600 ~ 0.6, protein expression was induced with 0.5 mM IPTG. The cultures were grown for 2 h at 37 °C. The affinity purification was performed as described for LolA-VC but at pH 6.0. After TEV cleavage the buffer was exchanged (20 mM MES pH 6, 0.2 M NaCl) and the protein solution was passed over the affinity column again. The flow through and wash fractions were collected, supplemented with 2 mM DTT and concentrated. The cleaved protein was further purified on a HiLoadTM 16/60 Superdex™200 prep-grade column (GE Healthcare) equilibrated with the same buffer. Fractions containing the peak of interest were concentrated (20 mM MES pH 6 and 0.2 M NaCl). The purity of the three proteins is presented in Fig. S6.

### Crystallization and structure determination

Crystallization screening was performed by the sitting-drop vapor-diffusion method at 20 °C in 96-well MRC-crystallization plates (Molecular Dimensions). Droplets of 0.2 µL protein were mixed with 0.1 µL of screening solutions from Molecular Dimensions using a Mosquito (TTP Labtech) pipetting robot.

LolA-PG was screened at 5 mg/mL and crystals were obtained in several conditions of the Morpheus screen. SeMet-labelled crystals (grown in 0.2 M Na formate, 0.2 M NH_4_acetate, 0.2 M Na citrate tribasic, 0.2 M Na/K tartrate, 0.1 M imidazole/MES pH 6.5, 40% PEG500mme, 20% PEG20000) were flash cooled in liquid nitrogen and stored until data collection. SAD data were collected at beamline P13 operated by EMBL Hamburg at the PETRA III storage ring (DESY, Hamburg, Germany). The structure of SeMet-LolA-PG, was solved with SAD-phasing using AutoRickshaw^38^. Density modification and automatic model building were performed using AutoRickshaw and ArpWarp^39^. The model was further built using rounds of manual building in COOT^40^ and refined to 1.7 Å using phenix.refine^41^.

LolA-VC was screened at 54 mg/mL. Small needle-like crystals were first obtained in #G6, MemGold Screen (0.1 M MES pH 5.0, 20 mM Zn-acetate and 18% PEG 8000); and optimized to 0.1 M MES pH 5.0, 20 mM Zinc-acetate, 20% PEG4000. The crystals were soaked for 30 s in mother liquor solution supplemented with 20% (v/v) PEG400 before they were flash cooled in liquid nitrogen. Data were collected at beamline ID23-2 operated by the European Synchrotron Radiation Facility (ESRF), Grenoble, France. The structure of LolA-VC was solved by molecular replacement using Phaser^42^ and a search model produced by Alphafold^22^ and was refined to 1.8 Å resolution.

LolB-VC was screened at 4.1 mg/mL. Crystals were obtained in #D7, MemGold2 (50 mM NaCl, 0.05 M MOPS pH 7.0, 19% w/v PEG6000). Micro seeding was performed, and crystals were optimized in condition #C7 (0.1 M KCl, 0.1 M Bis–Tris 6.0, 18% w/v PEG4000). The crystals were soaked in mother liquor solution supplemented with 20% v/v PEG400 before they were flash cooled. Data were collected at the BIOMAX beamline operated by MaxIV facility, Lund, Sweden The structure was solved by molecular replacement using Molrep in the CCP4 suite^43^ and the 1IWM structure^17^ as the search model and refined to 1.46 Å.

All diffraction images were automatically processed with XDS^44^ after data collection. Scaling was performed with Aimless^45^ from the CCP4 program suite^43^. All refinement was performed with phenix.refine^41^ in combination with rounds of manual building in COOT^40^. Figures were prepared using CCP4MG^46,47^. Data processing and refinement statistics are presented in Supplementary Table S1.

### Isothermal titration calorimetry experiments

ITC experiments were performed with MicroCal auto-iTC200 instrument at 25 °C (20 mM HEPES pH 7.5, 150 mM NaCl). Total injections were 20 of 2 µL except the first which was 0.4 µL and occurred every 150 s. Reference power was 10 µcal/s and initial delay of 60 s. For LolA and LolB interaction stirring speed was 300 rpm and for proteins and polymyxin B/A22 interactions stirring speed was 1000 rpm.

For LolA and LolB interactions, control experiments were performed by injecting LolA on buffer, buffer on LolB and buffer on buffer. For protein and polymyxin B/A22, control experiments were performed by injecting ligands with concentrations used in test, on buffer. Corresponding data was then subtracted from respective interactions as a linear fit. Raw data were fitted with MicroCal PEAQ-ITC analysis software using single site binding model for all the data sets.

## Supplementary Information

Supplementary Information.

## Data Availability

Data supporting the finding of this manuscript are available from the corresponding author upon request. Atomic coordinates for LolA-PG and LolA-VC (8CGM and 8CHX) and LolB (8CM1) have been deposited in the RCSB protein data bank.
