# Supplementary material for: A comparative analysis of lipoprotein transport proteins: LolA and LolB from Vibrio cholerae and LolA from Porphyromonas gingivalis
Source: Sci Rep. 2023 Apr 24;13:6605. doi: 10.1038/s41598-023-33705-y (PMC10126205; doi:10.1038/s41598-023-33705-y)
Supplement: Supplementary file 1 — Supplementary Information. [file 41598_2023_33705_MOESM1_ESM.pdf]

Supplementary information

A comparative analysis of lipoprotein transport proteins: LolA and LolB from *Vibrio cholerae* and LolA from *Porphyromonas gingivalis*

Deepika Jaiman[1,2], Raghavendra Nagampalli[1,2] and Karina Persson[1,2]*

[1]Umeå Centre for Microbial Research (UCMR).

[2]Department of Chemistry, Umeå University, 90187, Umeå, Sweden.

* Corresponding author:

Karina Persson
E-mail: karina.persson@umu.se
Tel: +46-90-7865926

.

Supplementary information

Supplementary figures:

a

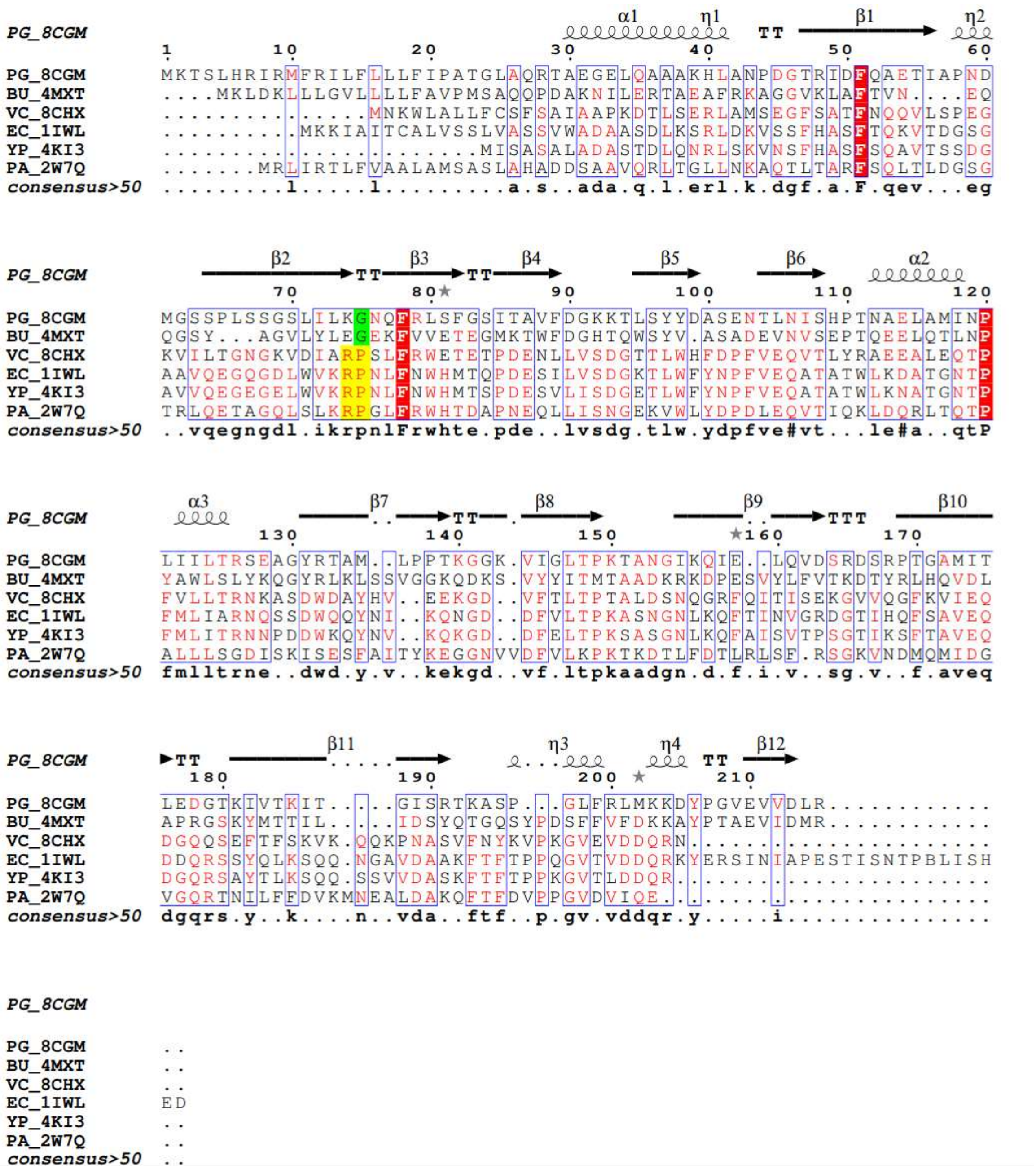

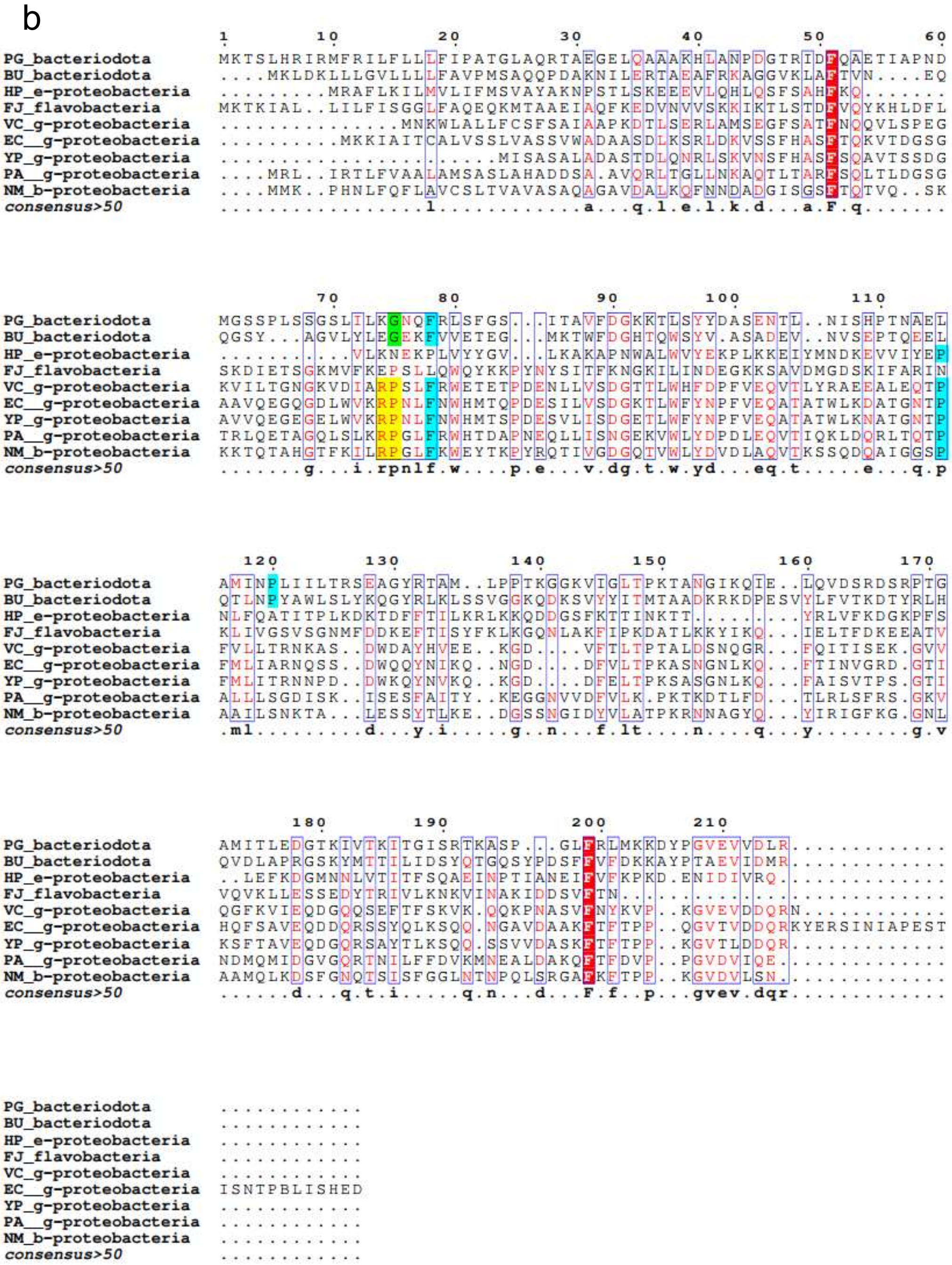

Supplementary Figure S1: A sequence alignment of LolA from different bacterial species. (a) A structure-based sequence alignment with LolA from *P. gingivalis* (PG_8CGM) on *B. uniformis* (BU_4MXT), *V. cholerae* (VC_8CHX), *E. coli* (EC_1ILW), *Y. pestis* (YP_4KI3), and *P. aeruginosa* (PA_2W7Q). Identical residues are shown in red and the Arg-Pro motif present in γ-proteobacteria is highlighted in yellow. The glycine located in the equivalent position in

bacteriodota is highlighted in green. Secondary structure elements, based on PG_8CGM, are indicated above the sequences. (b) LolA sequences from additional bacteria have been included in a sequence alignment. LolA protein sequences from *P. gingivalis* (PG), *B. uniformis* (BU), *Helicobacter pylori* (HP), *Flavobacterium johnsoniae* (FJ), *V. cholerae* (VC), *E. coli* (EC), *Y. pestis* (YP), *P. aeruginosa* (PA) and *Neisseria meningitidis* (NM) representing different phyla and classes. Identical residues are shown in red. Residues that were classed as identical in (a) are highlighted in blue. The Arg-Pro motif is highlighted in yellow and is found in γ-protobacteria and β-proteobacteria. The glycine in the same position found in *P. gingivalis* and *B. uniformis* is highlighted in green.

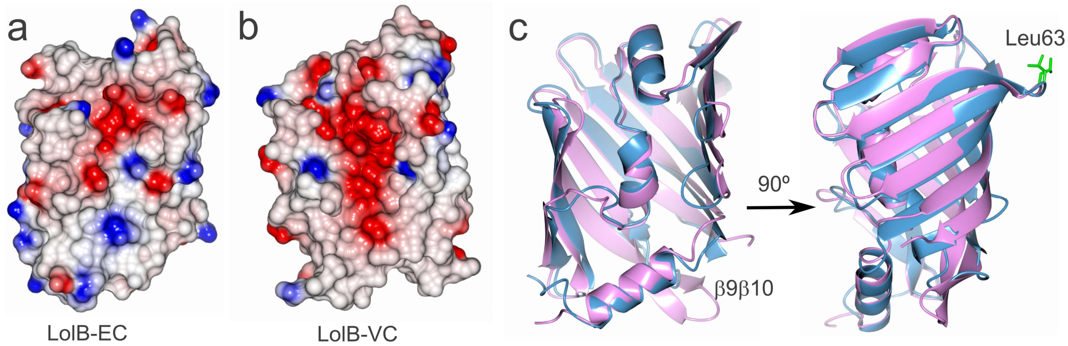

Supplementary Figure S2

Comparison between LolB-EC *and* LolB-VC. (a) Electrostatic surface representations show that (b) LolB-VC has a prominent electronegative patch present in the centre of the protein whereas the surface of LolB-EC has less charges. They also have different calculated isoelectric points, 5.1 and 8.7 respectively. (c) Superposition of LolB-VC (pink) and LolB-EC (blue) illustrating the same size and shape of the binding cleft. The longer β9β10-loop in LolB-VC and Leu63 (Leu68 in LolB-EC) are labelled.

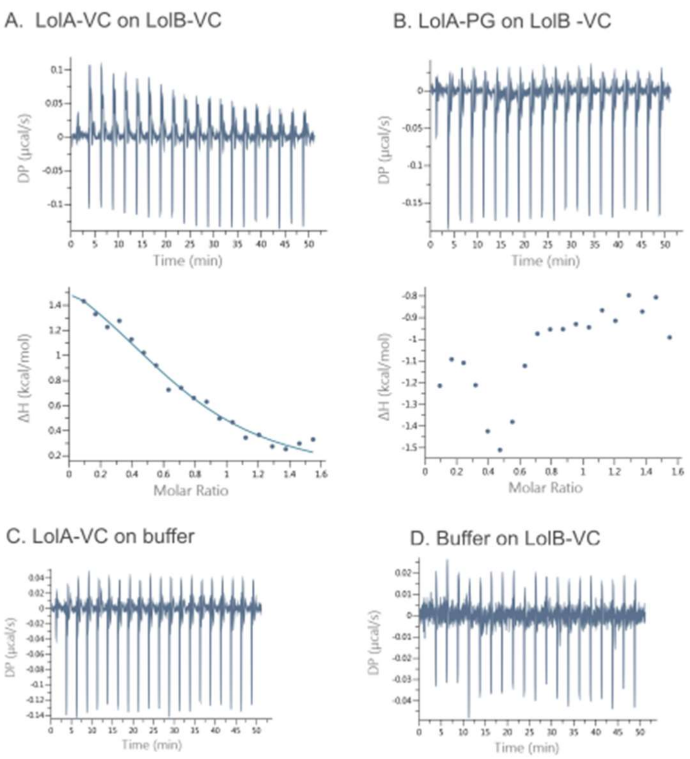

Supplementary Figure S3

The interaction between Lol proteins measured with ITC. (a) association of LolA-VC and LolB-VC, (b) the lack of association between LolA-PG and LolB-VC. For each titration, the thermogram is shown in the upper panel and the fitted curve of background-subtracted heats of injection in the lower panel. The shown thermograms which have been subtracted from heat of injection of LolA-VC to buffer. (c) The heat of injection of LolA-VC on buffer and (d) buffer on LolB-VC. Affinities and thermodynamic parameters are presented in **Table S2**.

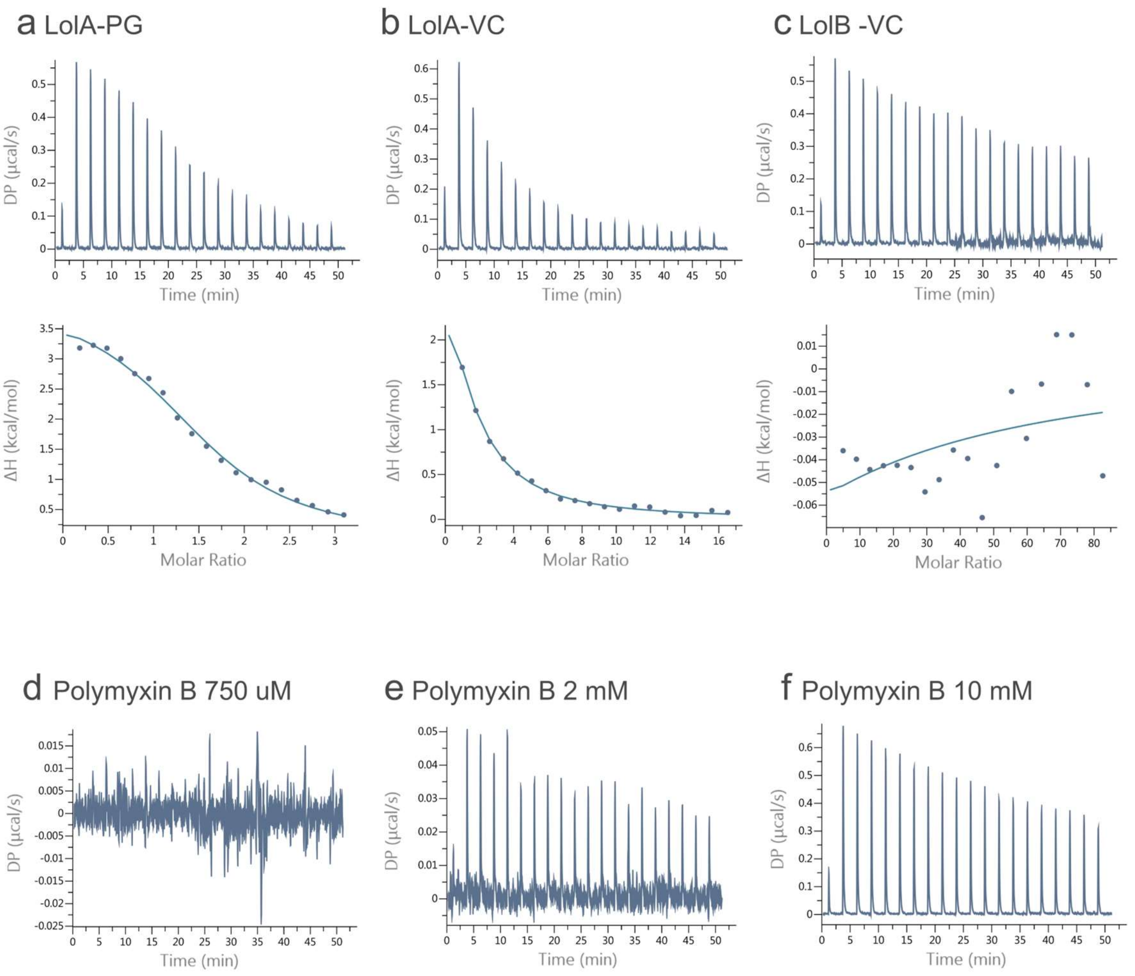

Supplementary Figure S4. The interaction between Lol proteins and polymyxin B. ITC profiles that illustrate the association between the antibiotic polymyxin B and (a) LolA-PG (b) LolA-VC and (c) LolB-VC. For each titration, the thermogram is shown in the upper panel and the fitted curve of background-subtracted heats of injection in the lower panel. The heat of injection of different concentrations of polymyxin B on buffer are shown in (d) 750 μM, (e) 2 mM and (f) 10 mM. Affinities and thermodynamic parameters are presented in Table S2 and S3.

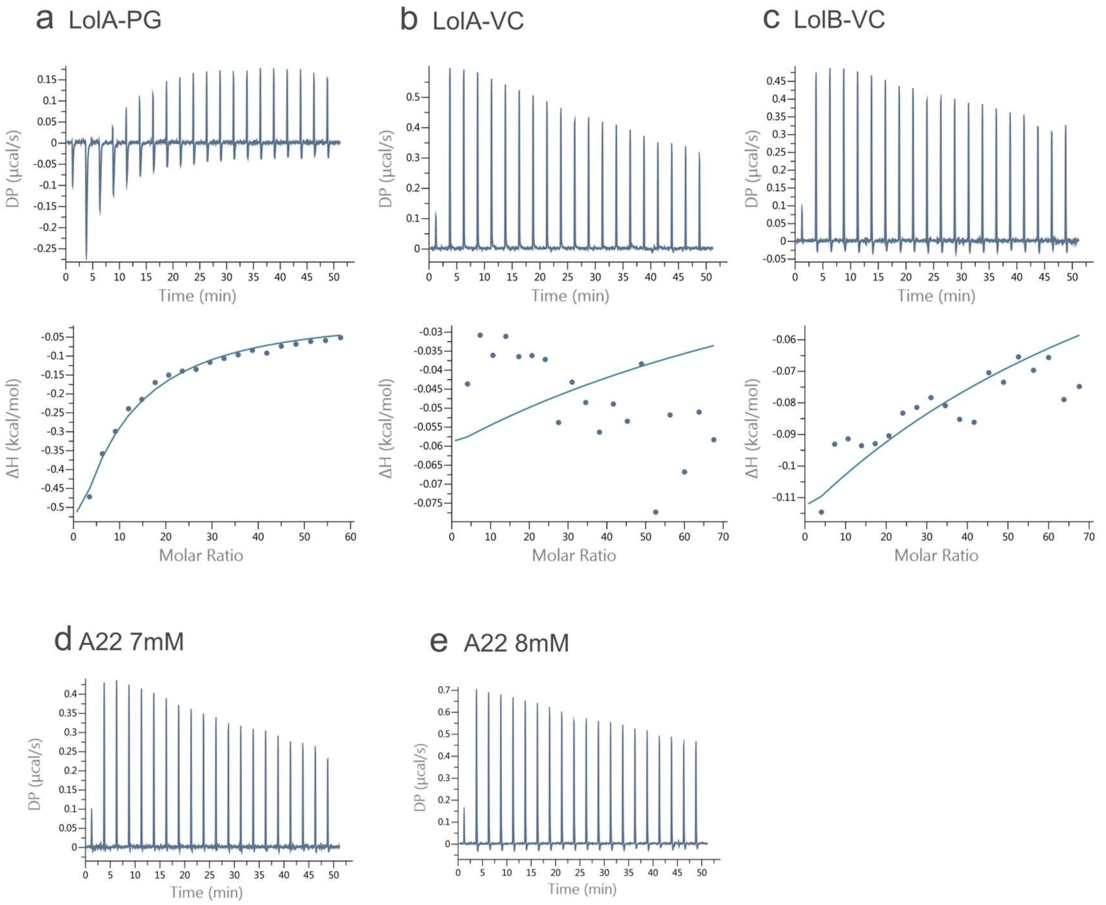

Supplementary Figure S5. The interaction between Lol proteins and the inhibitor A22. ITC profiles illustrating the association between A22 and (a) LolA-PG (b) LolA-VC and (b) LolB-VC. The thermogram is shown in the upper panel and the fitted curve of background-subtracted heats of injection in the lower panel. The heat of injection of two concentrations of A22 on buffer are shown in (d) 7 mM and (e) 8 mM. Affinities and thermodynamic parameters are presented in Table S2 and S3.

Supplementary information

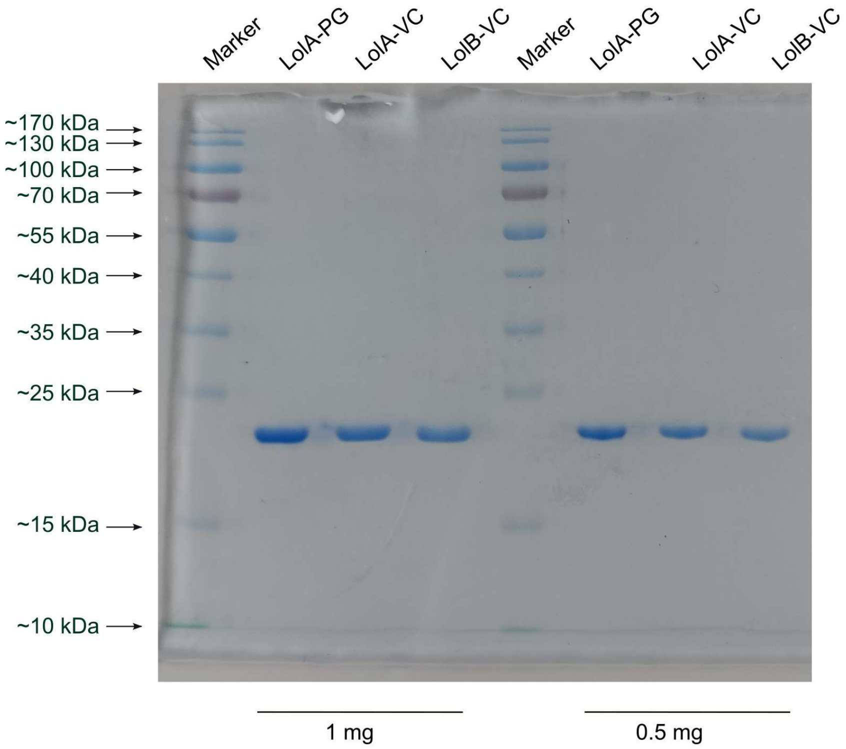

89

Supplementary Figure S6. SDS analysis of the purified proteins. Analysis of LolA-PG, LolA-VC, and LolB-VC. The molecular weights are 20.5, 20.7 and 21.8 kDa respectively with their His-tags removed. The samples have been analysed using both 1 mg and 0.5 mg sample.

Supplementary information

Supplementary Table S1: Data processing and refinement statistics

	LolA-PG-SeMet	LolA-VC	LolB-VC
**Data collection**			
Beamline	DESY, P13	ESRF, ID23-2	MaxIV, BioMAX
Wavelength (Å)	0.9762	0.8731	0.9762
Space group	$P2_12_12_1$	$P2_1$	$P\ 4_1\ 2_1\ 2$
Molecules in au	2	2	1
Cell dimensions			
a, *b*, *c* (Å)	48.8, 76,5. 99.4	46.5, 91.4, 48.5	113.4, 113.4, 34.2
α, β, γ (°)	90, 90, 90	90, 90.04, 90	90, 90, 90
Resolution (Å) *	49.7-1.7 (1.73-1.70)	45.8-1.80 (1.84-1.80)	32.7 - 1.46 (1.51-1.46)
R_{merge}	0.134 (1.299)	0.095 (0.675)	0.127 (2.410)
$I / \sigma I$	20.3(13.6)	15.3 (3.5)	19.6 (1.7)
Completeness (%)	100(100)	99.9 (100)	99.98 (99.97)
Redundancy	25.4(26.9)	9.9 (10.2)	25.7 (26.3)
CC1/2	0.999(0.865)	0.998 (0.871)	0.999 (0.681)
Molecules in a.u.	2	2	1
**Refinement**			
Resolution (Å)	49.7-1.70 (1.76-1.70)	42.8-1,80 (1.84.1.80)	35.9 - 1.46 (1.51 - 1.46)
No. reflections (work/test)	40327/1382	37460/3752	39342/1932
R_{work} / R_{free}	0.1792/0.2221	0.2134/0.2547	0.1658/0.1972
No. atoms	3369	3497	1692
Protein	2891	2921	1477
Ligand/ion	36	7	0

Water	442	562	192
B-factors (Å^2)			
Protein	26.7	25.7	21.7
Ligand/ion	44.0	28.9	0
Water	33.5	32.4	34.8
R.m.s. deviations			
Bond lengths (Å)	0.018	0.006	0.006
Bond angles (°)	1.509	0.860	0.950
PDB code	8CGM	8CHX	8CM1

*Values in parentheses are for the highest-resolution shell.

Supplementary Table S2: ITC Data for titrations with Kd in micromolar range

		Kd in µM	N sites	ΔH	ΔG	-TΔS
Cell<br>Syringe	100 µM LolB-VC<br>750 µM LolA-VC	27.5, 32.9<br>30.2 ± 3.8	0.72, 0.83<br>0.78 ± 0.08	2.0, 2.4<br>2.2 ± 0.3	-6.2, -6.1<br>-6.2 ± 0.1	-8.3, -8.5<br>-8.4 ± 0.2
Cell<br>Syringe	750 µM LolA-PG<br>100 µM LolB-VC	No binding				
Cell<br>Syringe	50 µM LolA-PG<br>750 µM<br>Polymyxin B	13.2, 13.7, 14.5<br>13.8 ± 0.7	1.56, 1.34, 1.39<br>1.43 ± 0.11	4.0, 4.8, 4.6<br>4.5± 0.4	-6.7, -6.6, -6.6<br>-6.6 ± 0.03	-10.6, -11.5, -<br>11.2<br>-11.1 ± 0.5
Cell<br>Syringe	25 µM LolA-VC<br>2 mM<br>Polymyxin B	61.1, 48.7, 58.2<br>56 ± 6.5	0.62, 1.83, 1.08<br>1.17 ± 0.61	11.2, 4.0, 6.7<br>7.2± 3.6	-5.8, -5.9, -5.8<br>-5.8 ± 0.06	-17, -9.9, -12.5<br>-13.2 ± 3.6
Cell<br>Syringe	25 µM LolA-PG<br>7 mM A22	732, 657, 654<br>681 ± 44	0.17, 0.17, 0.16<br>0.17 ± 0.005	-80.0, -80.0, -<br>80.0<br>-80.0 ± 0	-4.3, -4.3, -4.3<br>-4.3 ± 0	75.7, 75.7, 75.7<br>75.7 ± 0

The table presents each measurement and the average and standard deviation calculated on two or three replicates. *V. cholerae* (VC), *P. gingivalis* (PG). Mean ± Standard deviation in blue. ΔH, ΔG and –TΔS in kcal/mol. T = 25 ˚C

Supplementary Table S3: ITC Data for titrations with Kd in millimolar range

		Kd in mM	N sites	ΔH	ΔG	-TΔS
Cell<br>Syringe	25 µM LolB-VC<br>10 µM Polymyxin B	4.0 ± 43.4<br>0.4 ± 4.1	10 ± 890<br>10 ± 160	-0.92 ± 86.9<br>-0.04 ± 0.75	-3.3<br>-4.7	-2.4<br>-4.6
Cell<br>Syringe	25 µM LolA-VC<br>8 mM A22	13.7 ± 900<br>14.5 ± 1150	10 ± 10^{-4}<br>10 ± $1.3*10^{-4}$	-3.11 ± 3333<br>-3.47 ± 4580	-2.54<br>-2.51	0.57<br>0.97
Cell<br>Syringe	25 µM LolB-VC<br>8 mM A22*	9.05 ± 83.7<br>*$1*10^{-12} \pm 14*10^{-3}$*	10 ± 1160<br>*5.6 ± 1.5*	-4.19<br>*$7.7*10^{-2}$*	511<br>*$4.5*10^{-2}$*	1.4<br>*-16.4*

Values are shown with error values for individual experiments. A22 on LolB-VC * lost binding after subtraction of control experiment. The large deviations are due to the limited solubility of polymyxin A22.
